# Transcriptional profiling analysis of *Spodoptera litura* larvae challenged with Vip3Aa toxin and possible involvement of trypsin in the toxin activation

**DOI:** 10.1038/srep23861

**Published:** 2016-03-30

**Authors:** Feifei Song, Chen Chen, Songqing Wu, Ensi Shao, Mengnan Li, Xiong Guan, Zhipeng Huang

**Affiliations:** 1 Key Laboratory of Biopesticide and Chemical Biology, Ministry of Education, Fujian Agriculture and Forestry University, 350002 Fuzhou, Fujian, People’s Republic of China

## Abstract

Vip proteins, a new group of insecticidal toxins produced by *Bacillus thuringiensis*, are effective against specific pests including *Spodoptera litura*. Here, we report construction of a transcriptome database of *S. litura* by *de novo* assembly along with detection of the transcriptional response of *S. litura* larvae to Vip3Aa toxin. In total, 56,498 unigenes with an N50 value of 1,853 bp were obtained. Results of transcriptome abundance showed that Vip3Aa toxin provoked a wide transcriptional response of the *S. litura* midgut. The differentially expressed genes were enriched for immunity-related, metabolic-related and Bt-related genes. Twenty-nine immunity-related genes, 102 metabolic-related genes and 62 Bt-related genes with differential expression were found. On the basis of transcriptional profiling analysis, we focus on the functional validation of trypsin which potentially participated in the activation of Vip3Aa protoxin. Zymogram analysis indicated that the presence of many proteases, including trypsin, in *S. litura* larvae midgut. Results of enzymolysis *in vitro* of Vip3Aa by trypsin, and bioassay and histopathology of the trypsin-digested Vip3Aa toxin showed that trypsin was possibly involved in the Vip3Aa activation. This study provides a transcriptome foundation for the identification and functional validation of the differentially expressed genes in an agricultural important pest, *S. litura*.

*Spodoptera litura* (Fab.) (Noctuidae: Lepidoptera) is notorious as one of the most destructive pests of agricultural crops, including more than 120 host plants globally^1^,^2^. It is a serious pest of soybean, cotton, groundnut, taro, tobacco, flax and some important vegetables, including potato, eggplants, cabbage, capsicum, brinjal, okra, brassica and cucurbits^3^,^4^. The species is responsible for huge yield losses annually in many crops due to its vigorous defoliation^5^. It has shown resistance to numerous synthetic insecticides, which has led to frequent control failure in crops^6^,^7^,^8^. As a consequence, considerable attention has been paid to biological insecticides as alternatives for controlling pests.

*Bacillus thuringiensis* is the most widely used commercial biopesticide with wide pathogenicity against numerous species^9^. At present, the Cry toxins produced by this bacterium are the most well-known biocontrol agents^10^. In addition to Cry toxins, some *B. thuringiensis* strains produce other insecticidal proteins during the vegetative growth phase, named vegetative insecticidal proteins (Vip)^11^,^12^. Vip toxins share no sequence homology with Cry toxins^13^ and have been classified into four subfamilies, Vip1, Vip2, Vip3 and Vip4, according to their sequence homology (http://www.lifesci.sussex.ac.uk/home/Neil_Crickmore/Bt/vip.html). To date, most studies on Vip toxins have been carried out with Vip3A. Since the discovery of the first Vip toxin, a great number of *B. thuringiensis* collections have been screened for new *vip* genes^14^,^15^,^16^,^17^ and the insecticidal activities of this kind of toxins have also been tested^10^,^18^,^19^,^20^. Vip3A toxins have already been incorporated into crops for the control of Lepidopteran pests^21^, but the mode of action of the toxin is still unclear. It is commonly accepted that, similar to Cry toxins, Vip3A toxins need to be activated by proteolysis and then the active forms bind to specific receptors on the midgut brush border membrane which eventually lead to pore formation and cell lysis^22^,^23^,^24^. Vip3A toxins, however, differ in their mode of action from Cry toxins in several key steps, especially in the binding step^23^,^24^,^25^. The binding sites of Vip toxins and Cry toxins in *Helicoverpa armigera*, *Spodoptera frugiperda* and *Agrotis segetum* have been shown to be different^23^,^26^,^27^,^28^. This contribute to the insecticidal activity of *B. thuringiensis* against insects, and is especially useful for the management of insect resistance to Cry toxins.

Recently, proteomic analysis and transcriptional profiling analysis have been used to investigate the defense response of some insects to Cry toxins^29^,^30^,^31^,^32^,^33^, and to gain insight into the mode of action of Cry toxins^34^,^35^,^36^,^37^. However, there is no report about the defense response of *S. litura* to Vip toxins by transcriptional profiling analysis. In the present study, the complete transcriptome information of *S. litura* was obtained and a genome-wide high-throughput transcriptome sequencing was used for comparison of Vip3Aa-treated and non-treated larvae of *S. litura*. The obtained transcriptome sequences provided genetic information to investigate the complex response of *S. litura* larva to Vip3Aa toxin by monitoring gene expression levels. Selected genes with different expression patterns were further validated by qRT-PCR. On the basis of transcriptional profiling analysis, the function of trypsin was validated by enzymolysis *in vitro*, bioassay and histopathology. Transcriptional profiling could provide a better understanding of the complex response of the *S. litura* midgut to Vip3Aa toxin and laid a foundation for further verifying the functions of the genes with different expression of *S. litura*, which are useful for future biocontrol strategies.

## Results

### Sequence Analysis and *De Novo* Assembly

A complete transcriptome was generated to obtain as many functional gene transcripts as possible. After removing low-quality sequences and adaptor sequences, a total of 56,108,398 clean reads were generated for control *S. litura* larvae midgut and 56,586,122 for Vip3Aa-treated larvae midgut. The quality value ≥30 with more than 89.79% bases for each sample suggested the sequencing was highly accurate. The GC content was about 48%, and 83.66–89.66% of the reads were mapped to unique sequences. After assembly using the Trinity method, 56,498 unigenes with an N50 value of 1,853 bp and a mean length of 827.6 bp were obtained (Table 1). Unigenes with a length between 200 and 300 bp represented the highest proportion.

### Functional Annotation

**Figure 1:**
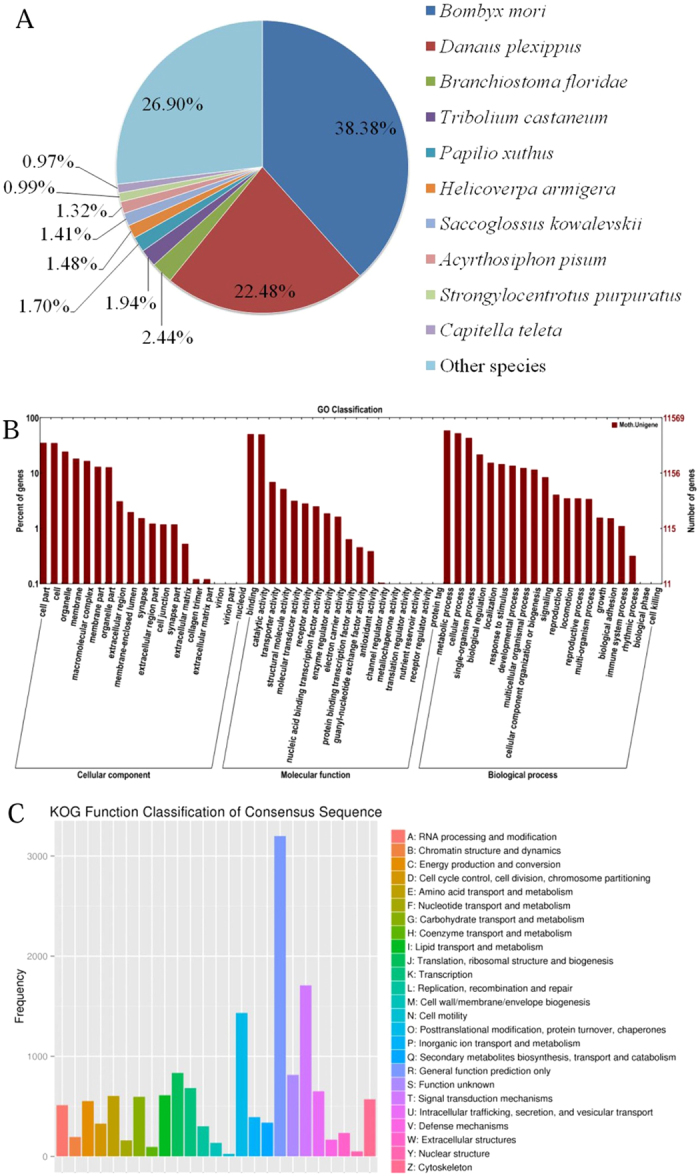
Overview of *S. litura* annotations. (**A**) Species distribution of the BLAST_X_ results. (**B**) GO categories of all unigenes and DEGs. (**C**) euKaryotic orthologous Groups (KOG) classification.

The unigenes were annotated against the following databases: NCBI nonredundant protein database (Nr), SwissProt database, Protein family (Pfam), euKaryotic Orthologous Groups of proteins (KOG), Kyoto Encyclopedia of Genes and Genomes (KEGG) and Gene Ontology (GO) database. A total of 21,701 unigenes (38.41%) were successfully annotated, including 20,541 (36.35%) present in the Nr database, 13,126 (23.23%) in the SwissProt database and 6,296 (11.14%) in the KEGG database. Nr database queries also revealed the distribution and frequency of the species in the homology search (Fig. 1A). A high percentage of *S. litura* sequences closely matched sequences of silkworm (*Bombyx mori*, 38.38%) and monarch butterfly (*Danaus plexippus*, 22.48%).

The GO distributions of the unigenes were classified into three categories (57 subcategories): cellular components (19 subcategories), molecular functions (18 subcategories), and biological processes (20 subcategories) (Fig. 1B). In the category of cellular components, the clusters relating to “cell part” with 4,096 genes and “cell” with 4,068 genes were enriched. In the category of molecular functions, the clusters of “binding” and “catalytic activity” were highly represented (5,091 and 5,841 genes, respectively). In the category of biological processes, “metabolic processes”, “cellular processes” and “single-organism processes” were large compared to other subcategories with 6,855, 6,120 and 5,041 genes, respectively. In addition, the orthologous functions of the identified unigenes were defined using the KOG database (Fig. 1C). The “general function prediction” cluster (3,198, 21.07%) occupied the highest proportion, followed by “Signal transduction mechanisms” (1,708, 11.25%) and “Posttranslational modification, protein turnover, chaperones” (1,432, 9.43%). The two smallest groups were “Cell motility” and “Nuclear structure”.

### Complex response of *S. litura* midgut to Vip3Aa toxin

The hierarchical cluster generated from transcriptome data was used to determine the profiles of the differentially expressed unigenes between control and Vip3Aa-treated *S. litura* (Figure S1A in Supplementary Material). The overall pattern of transcript changes after Vip3Aa ingestion were shown by gathering the same or similar expression unigenes in the same cluster. A total of 1,253 significantly altered unigenes was detected between control and Vip3Aa-treated *S. litura* samples (Figure S1B in Supplementary Material). The number of up-regulated genes (742) was greater than the number of down-regulated genes (511), which is similar to the report for *S. exigua*^38^.

In order to detect the complex response of the *S. litura* midgut to Vip3Aa toxin, GO annotation, KEGG database and BLAST searches were combined to identify differentially expressed in immunity-related, metabolic-related and Bt-related genes. In total, 29 differentially expressed immunity-related genes were identified (Table S1 in Supplementary Material), including 12 genes for recognition, 11 genes for signal transduction and melanization processes, 3 genes for antimicrobial effector and 3 genes for other immune-related proteins. The majority of these genes were up-regulated after Vip3Aa feeding except PGRP (peptidoglycan recognition protein) and superoxide dismutase. Differentially expressed metabolic genes from the transcriptome data were identified and listed in Figure S2 in Supplementary Material. In total, 102 metabolic genes were differentially expressed between control and Vip3Aa-treated *S. litura*, and participated in one or more pathways. Five types of mainly basal metabolic systems were identified, including amino acid metabolism, metabolism of cofactors and vitamins, lipid metabolism, xenobiotics biodegradation and metabolism, and carbohydrate metabolism. Bt-related genes mainly included genes encode proteases (51 genes with differential expression, part of them was shown in Table 2) participated in the activation or degradation of Vip protoxin and unigenes homologous to the potentially Bt toxin receptors (11 genes with differential expression, shown in Table S2 in Supplementary Material). Other genes with high level regulation after Vip3Aa feeding were shown in Table S3 in Supplementary Material.

**Figure 2:**
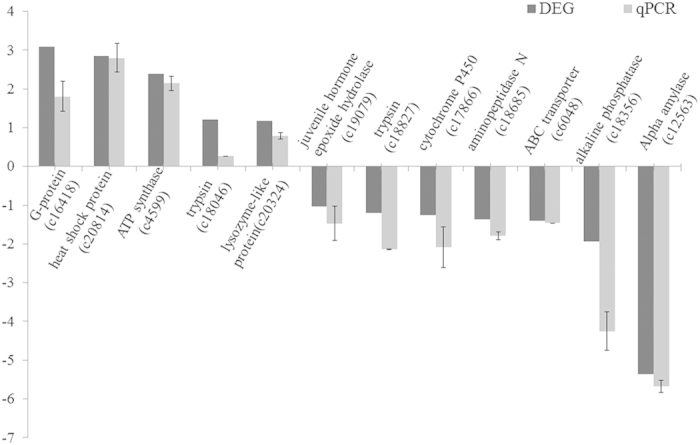
Validation of transcriptome data by qRT-PCR. The means of at least three biological replicates are presented as log2FC ± SE.

To validate the transcriptome data, qRT-PCR analysis was performed for 12 differentially expressed genes including five up-regulated and seven down-regulated unigenes (underlined unigenes in Table 2, Tables S1–S3 in Supplementary Material). The regulated genes selected for confirmation included immune-related unigenes, Bt-related unigenes and other significantly differentially expressed unigenes. The qRT-PCR validation results were presented as fold-changes normalized to the *β-actin* gene. A comparison of the mean values of qRT-PCR results and the DEGs is summarized in Fig. 2. The consistent expression trend in qRT-PCR results and the original DEGs indicates the reliability of gene expression profiling from transcriptome analysis.

### Identification of serine protease genes of *S. litura* midgut potentially in response to the ingestion of Vip3Aa toxin

Activation or degradation of *B. thuringiensis* protoxin is a primary factor influencing its toxicity after ingestion. Unigenes encode proteins participated in the activation or degradation of Vip protoxin were extracted from transcriptome data for the identification of the differentially expressed genes. Serine proteases were found to be constituted the most abundant differentially expressed group of transcripts when *S. litura* larvae feeding with Vip3Aa toxin. Most of the serine proteases genes in midgut, which were up-regulated relative to whole larvae (Table 2, left), were found differentially expressed when *S. litura* larvae feeding with Vip3Aa toxin (Table 2, right). This result provided a preliminary proof that serine protease maybe involved in the solubilization of Vip3Aa toxin. Due to the complexity of serine protease family, we select one of serine protease, trypsin, for the further validation.

### Validation of trypsin possibly involved in the Vip3Aa toxin activation

**Figure 3:**
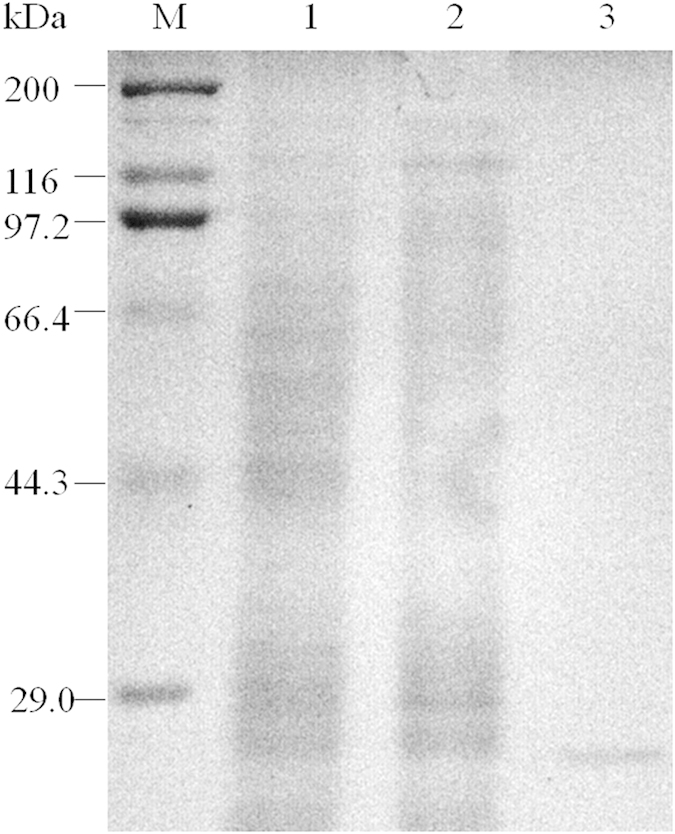
Zymogram analysis of *S. litura* larvae midgut proteases. Midgut soluble proteases (lane 1), midgut membrane-bound proteases (lane 2) and trypsin (lane 3) were separated by 12% SDS-PAGE and their activity revealed using casein as substrates.

Using a general substrate, casein, as substrate, zymogram analysis was performed with the soluble proteases and membrane-bound proteases prepared from *S. litura* larval midgut and commercial trypsin. Trypsin can degrade casein into a band of 25.7 kDa (Fig. 3, lane 3). Zymogram of the soluble proteases and membrane-bound proteases showed the presence of many active proteases including trypsin (Fig. 3, lane 1 and 2).

**Figure 4:**
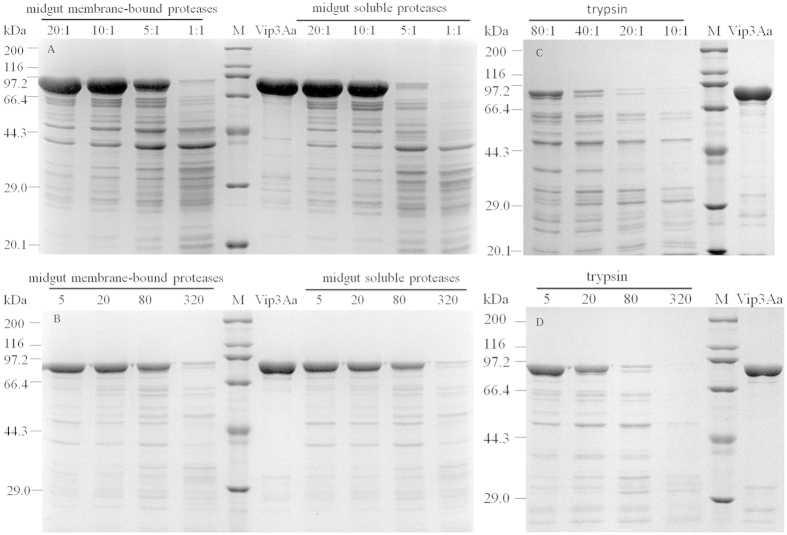
Proteolysis processing of Vip3Aa toxin by *S. litura* midgut proteases and commercial trypsin. (**A**,**C**) Digestion of Vip3Aa toxin by different concentrations of proteases. Vip3Aa/proteases (midgut soluble, membrane-bound proteases or trypsin) ratio was showed in the figure (μg/μg). The toxins digestion with different incubation times were conducted for 5, 20, 80 and 320 min at the optimal activation concentration (**B**,**D**). Molecular mass markers (M) in kDa are indicated in the middle of the figure.

After confirmation the presence of trypsin in *S. litura* larval midgut, We further investigated enzymolysis *in vitro* of Vip3Aa by the soluble proteases and membrane-bound proteases prepared from *S. litura* larval midgut and trypsin with different concentrations and incubation times. Although their SDS-PAGE profiles were slightly different, the bands between 66.4 kDa and 29 kDa are similar especially in five major bands about 64, 62, 59, 45 and 39 kDa (Fig. 4A,C). The SDS-PAGE profiles obtained with different incubation times indicated that Vip3Aa proteolysis occurred rapidly as detectable small fragments was found after 5 min (Fig. 4B,D).

**Figure 5:**
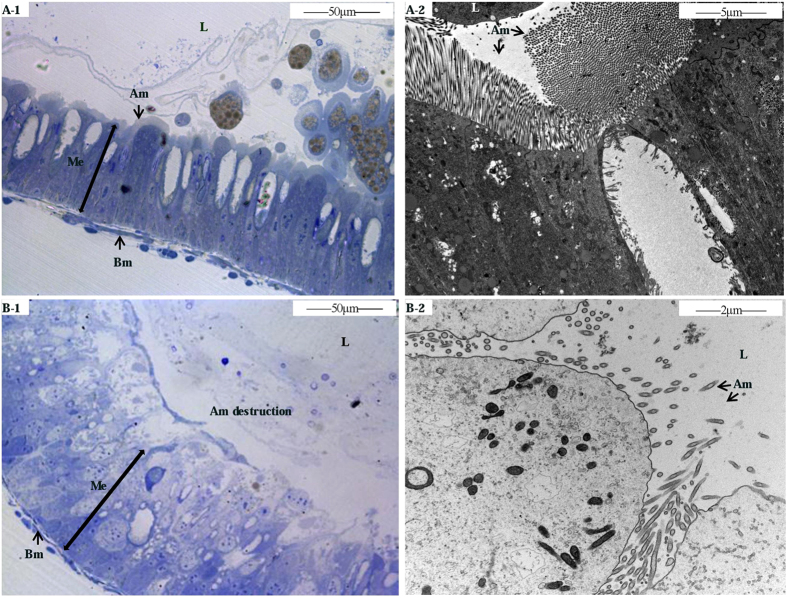
Histopathological effects of the trypsin-activated Vip3Aa toxin on *S. litura* larvae midgut. general aspects of the midgut (**A**) and midgut of larvae fed with trypsin-activated Vip3Aa toxin (**B**). Me midgut epithelium, Am apical membrane, Bm basement membrane, L lumen.

The insecticidal activity and histopathology of the trypsin-digested Vip3Aa toxin were also assayed. The digested products show high toxicity to first instar *S. litura* larvae with an LC_50_ of 77.052 ng/cm^2^ artificial diet with 95% confidence limits of (66.304–90.649 ng/cm^2^). Compared to control untreated-midgut, the observations of midgut cross sections showed wide damage to *S. litura* larvae midgut epithelium induced by the digested products. The histopathological changes of larvae fed with the trypsin-digested Vip3Aa toxin mainly included vacuolization of the cytoplasm, cells swelling and brush border membrane destruction (Fig. 5B), while control untreated-midgut showed a well-preserved layer of epithelial cells with unaffected apical microvilli membrane (Fig. 5A). These results demonstrated that trypsin is possibly involved in the activation of Vip3Aa toxin.

## Discussion

Recently, transcriptome sequencing has become a crucial research method on account of its peculiarity of low cost and high throughput^39^. We presented here the complete transcriptome information of *S. litura*, and a complex response of the *S. litura* larval midgut to Vip3Aa toxin. Due to the lack of a reference genome of *S. litura*, the unigenes were identified and annotated using public protein databases including the Nr, GO, KEGG, KOG, Swiss-Prot and Pfam databases. Over the past few years, GO terms have been utilized for the functional annotation and visualization of unigenes. A framework for categorizing genes was provided, namely biological process, molecular function, and cellular compartment. In the present study, 11,569 unigenes were assigned to one or more ontologies. KEGG analysis was utilized for demonstrating the various biological pathways^40^, and 6,296 unigenes were assigned to KEGG pathways. Collectively, the data portrayed a perspective of *S. litura* transcriptome information and also the differential expression of *S. litura* genes induced by Vip3Aa toxin treatment.

Previous studies have demonstrated that Cry intoxication of insects induced differential gene expression related to immunity and metabolism^31^,^41^. As mentioned above, the majority of immune-related genes we observed were up-regulated after Vip3A intoxication. In the insect innate immune response, successful recognition of a potential pathogen is the initial process. It is currently known that this recognition is mediated by pattern recognition proteins (PRPs)^42^,^43^. However, after Vip3Aa feeding, we found most of PRPs such as hemolin, β-1,3-glucan recognition proteins (βGRPs), C-type lectins(CTLs), scavenger receptors (SCRs) were all over-expressed, but peptidoglycan recognition proteins (PGRPs) were down-regulated. The silimar result had also been found in *S. exigua* after Vip3A feeding^38^. It can be speculated that PRPs probably have other functions, besides the mediation of pathogen recognition. In addition, melanization occurs regularly in the insect midgut and two up-regulated genes of the melanization cascade were observed after Vip3A intoxication. After recognition and melanization of the invasive signals, the immune system of insects mainly relies on signaling pathways to produce the effector molecules. The main signal transduction pathways involved in insect immunity are the janus kinase/signal transduction and activator of transcription (JAK/STAT), Mitogen Activated Protein Kinase (MAPK), Toll, immune deficiency (IMD), and c-jun Nterminal kinase (JNK) pathways^38^,^44^. We identified signal transduction-related genes related to JAK/STAT, Toll and MAPK pathways. The final effectors insects produce upon infection are induced following successive signal recognition, melanization, and transduction. Part of the synthesized effector molecules play direct roles in elimination of infections, phenol oxidase-dependent melanization and other immune-related mechanism^48^. Vip3Aa feeding also revealed up-regulation of other associated immune-related genes, such as Hdd family.

The basic metabolism response is very important for maintaining the normal physiological activities of organisms. Analysis of the differential expression of genes related to basal metabolic pathways between control and Vip3Aa-treated *S. litura* contributes to understanding the interaction between insect and toxin. In this study, we found that the number of unigenes and transcripts related to metabolism was significantly changed after infection. The results shown in Figure S2 (Supplementary Material) indicated that Vip3Aa affected the basal metabolic system-related genes of *S. litura* in many pathways, including glycandegradation, oxidative phosphorylation, and fatty acid biosynthesis, leading to over or reduced expression. However, the mechanisms of the basal metabolic pathways modulation are not exactly known.

After Vip protoxin is ingested by insect, it is processed to an active toxin which can bind with insect gut receptors^45^. Previous studies in some lepidopteran insects had reported that a 62 kDa fragment produced by the Vip3A protoxin enzymolysis is the active form of this protein^20^,^22^,^24^, and that trypsin are responsible for the activation of Cry protoxin^46^. However, for *S. litura*, there is so far no report about the activation process of Vip3Aa toxin and the role of trypsin in the Vip3Aa activation. By means of transcriptome analysis, We found that 34 serine proteases genes in midgut were up-regulated relative to whole larvae (Table 2, left), and that 14 genes were up-regulated and 7 genes were down-regulated in Vip3Aa-treared midgut compared to non-treated midgut (Table 2, right). The significant changes in the gene expression after Vip3Aa toxin ingestion suggested that the candidate genes may involved in the process of toxin activation or degradation. Zymogram of the soluble proteases and membrane-bound proteases showed the presence of many active proteases including trypsin (Fig. 3). In enzymolysis *in vitro* of Vip3Aa toxin by trypsin, a 62 kDa fragment which is considered as the active form in some other lepidopteran insects also appeared. The trypsin-digested Vip3Aa toxin is high toxicity to first instar *S. litura* larvae and can cause wide damage to midgut epithelium. These result demonstrated that trypsin is possibly involved in the activation of Vip3Aa toxin, which is the same as the report in Cry toxin.

Although previous studies about some Lepidopteran pests have shown that the binding sites of Vip toxins and Cry toxins are different, some general characterized receptors genes for Cry toxin were found to be differentially expressed after Vip3Aa ingestion (Table S2). In the Cry toxins pore-formation model of Lepidoptera, the activated toxin first binds to cadherin to form toxin oligomers, which may involved in alkaline phosphatases (ALPs) and N-aminopeptidases (APNs). Two differentially expressed cadherin-like transcript were found after Vip3Aa ingestion, but it is unclear why the pattern of transcript changes were different with one up-regulated and one down-regulated. Unigenes homologous to ALPs and APNs were found down-regulated, whereas G-proteins were up-regulated. The changes of ALPs and G-proteins are the same as the report in *S. exigua*^38^. Further studies are needed to know their functions.

In summary, in this study, the complete transcriptome information of *S. litura* has been obtained, and the overall response of the *S. litura* midgut to Vip3Aa toxin has been described for the first time. On the basis of transcriptional profiling analysis, we further investigated the *in vitro* activation process of Vip3Aa toxin, and demonstrated that trypsin is possibly involved in the Vip3Aa toxin activation. The obtained transcriptome data provides a foundation for the identification and functional validation of the differentially expressed genes of *S. litura* after Vip3Aa toxin feeding.

## Materials and Methods

### Insects and toxin

The colony of *S. litura* used in the experiments was purchased from Ke Yun Biology co. (China) and reared on artificial diet under the following conditions: temperature 27 ± 2 °C, relative humidity 55 ± 5% and photoperiod 16:8 h (light/dark).

The *vip3Aa* gene (GenBank Accession No. AF500478) was from *B. thuringiensis* WB5, a native strain isolated from soil collected in Wuyi mountain (China). The pCzn1-*vip3Aa*, a recombinant plasmid constructed in our previous study, was transformed into *E. coli* BL21 and the transformants were induced with 0.2 mM isopropyl-D-thiogalactopyranoside (IPTG) at 16 °C for 4 h. After centrifugation at 1,2000 g at 4 °C for 10 min, the pellet was resuspended and sonicated on ice, then centrifuged at 1,2000 g at 4 °C for 10 min. The supernatant was purified using Ni-IDA-Sepharose affinity column, dialyzed with PBS buffer and stored at −20 °C for the feeding experiments. The concentrations of Vip3Aa toxin were measured according to the Bradford method^47^.

### Treatment of *S. litura* larvae with Vip3Aa

Third instar larvae of *S. litura* were selected for the feeding experiments. The larvae were reared on artificial diet containing Vip3Aa toxin with an LC_50_ of 200 ng/cm^2^, which resulted in a growth inhibition but did not cause visible death of the larvae for 24 hours. Larvae treated with PBS buffer were used as negative control. All treated larvae were incubated for 24 h at 27 ± 2 °C, 55 ± 5% RH, and L16:D8 h. Ten larvae were used for each treatment, and at least five larvae that had fed (as determined by observing the food bites) were dissected to obtain midgut tissues for further processing. Both treatments were performed in triplicate.

### RNA isolation, cDNA library construction, and Illumina sequencing

Total RNA was extracted from the whole body and midgut of *S. litura* respectively, using the TRIzol Reagent (Invitrogen) following the protocols provided by the manufacturers. The integrity, quantity and quality of total RNA were determined using the RNA Nano 6000 Assay Kit of the Agilent Bioanalyzer 2100 system (Agilent Technologies, CA, USA), the Qubit^®^ RNA Assay Kit in Qubit^®^2.0 Flurometer (Life Technologies, CA, USA) and the NanoPhotometer^®^ spectrophotometer (IMPLEN, CA, USA).

The mRNA was enriched and isolated from total RNA using poly-T oligo-attached magnetic beads. Fragmentation was carried out using a RNA fragmentation kit, followed by synthesis of first and second strand cDNA using M-MuLV Reverse Transcriptase (RNase H-), DNA polymerase I and RNaseH. The cDNA fragments were end-repaired and ligated to a NEBNext Adaptor after adenylation of the 3′ ends, then purified with the AMPure XP system (Beckman Coulter, Beverly, USA) to create a cDNA library. Library quality was assessed on the Agilent Bioanalyzer 2100 system. After clustering, the products were sequenced on an Illumina Hiseq 2500 platform.

### *De Novo* Assembly and Data analysis

The raw data were first filtered by removing the adaptor sequences, reads containing ploy-N and low quality reads. Then the quality value and base distribution of the raw data were examined by calculating Q20, Q30, GC-content and sequence duplication level of the clean data. *De novo* assembly of the mRNA-seq reads was accomplished using Trinity^48^ with K-mer = 25. Annotations of the unigenes were performed by a BLASTx search against the Nr, Swiss-Prot, KOG, KEGG databases and Pfam, and by a Blast2GO search against the GO database. Gene expression levels were calculated by RSEM^49^. Differential expression of the unigenes was performed using the DEGseq^50^ R package. False discovery rate (FDR) <0.01 and log2 ratio ≥1 were set as thresholds for identifying significant differential expression between control and Vip3Aa treated larvae.

### Quantitative real-time PCR (qRT-PCR) validation

To confirm the data, a subset of differentially expressed genes was validated by quantitative real time PCR (qRT-PCR). Total RNA from each sample was extracted as described above. Reverse transcription was performed using a PrimeScript RT reagent kit with the gDNA Eraser (TOYOBO). Quantitative real-time PCR was conducted on the CFX96 Real-Time System (Bio-Rad, Hercules, CA, USA) using the SybrGreen method with Premix Ex Taq II (Takara, Kyoto, Japan). The selected genes were verified with the following cycling conditions: 95 °C for 30 s, followed by 40 cycles of 95 °C for 30 s, 60 °C for 35 s. The melting curve analysis was used to analyze the specificity of the qPCR product. The sequences of the primers used are listed in Table S4 in Supplementary Material. A β-actin served as an internal control. The relative gene expression values were calculated using the 2^−△△Ct^ method^51^.

### Preparation of *S. litura* larvae midgut proteases and Zymogram analysis

Third instar *S. litura* larvae were collected and anesthetized on ice for 30 min, then dissected the midgut. The soluble fraction and membrane fraction extracts of the midgut were obtained by centrifugation after homogenating^52^, then separated by SDS-PAGE. After washed in turns with Triton X-100 (2.5%) and water, the gel was incubated with 2% casein in 50 mM Na_2_CO_3_ buffer (pH 9.6) at 37 °C for 3 h. Then the protease activity was visible after Coomassie staining.

### Enzymolysis *in vitro* of Vip3Aa toxin by commercial trypsin and *S. litura* midgut proteases

The purified Vip3Aa toxin was mixed with each of different concentrations of *S. litura* larvae midgut proteases and commercial trypsin (Sigma) in 100 mM PBS buffer (pH 7.2, similar to *S. litura* midgut) and incubated for 1 h at 30 °C. To investigate the enzymolysis kinetic of the purified Vip3Aa toxin, it was mixed with each protease as mentioned above and incubated for diverse incubation times (between 5 and 320 min) at 30 °C. The Vip3Aa/protease ratio was the optimal activation concentration. The proteolytic reaction was stopped by the addition of Laemmli sample buffer (5×) followed by heat denaturation. Samples were separated by 12% SDS–PAGE and stained with Coomassie blue.

### Bioassay and histopathology observation of the trypsin-digested Vip3Aa toxin

The toxicity to first instar *S. litura* larvae was tested by feeding with five different concentrations of the trypsin-digested Vip3Aa toxin in artificial diet. PBS buffer was used as a negative control. All treatments were in sextuplicate with ten larvae per replicate. The bioassays were performed in a growth chamber under the following conditions: temperature 27 ± 2 °C, relative humidity 55 ± 5% and photoperiod 16:8 h (light/dark). The mortality of each treatment was recorded every other day. Median lethal concentrations (LC_50_) and ninety percent lethal concentrations (LC_90_) were estimated from mortality data by probit analysis (DPS software).

Third instar *S. litura* larvae were starved for 2 h, and then fed with artificial diet supplemented with the trypsin-digested Vip3Aa toxin (200 ng/cm^2^ of diet) for 24 h. Artificial diet supplemented with PBS buffer was used as a negative control. After chilled on ice for 30 min, the midguts were excised and fixed in 2.5% glutaraldehyde and 1% osmic acid stationary liquid. Tissue were dehydrated using increasing ethanol concentrations, then rinsed in 100% acetone and fixed by embedding and curing. Images were captured with transmission electron microscopy (TEM, JEM-2010(HR)).

## Additional Information

**How to cite this article**: Song, F. *et al*. Transcriptional profiling analysis of *Spodoptera litura* larvae challenged with Vip3Aa toxin and possible involvement of trypsin in the toxin activation. *Sci. Rep*. **6**, 23861; doi: 10.1038/srep23861 (2016).

## Supplementary Material

Supplementary Information

## Figures and Tables

**Table 1:** Unigene assembly of *S. litura* transcriptome.

Length range (nt)	Unigene count	Percentage
200–300	23,039	40.78%
300–500	13,460	23.82%
500–1000	8,472	15.00%
1000–2000	5,580	9.88%
2000+	5,945	10.52%
Total Number	56,498	
Total Length	46,757,968	
N50 Length	1,853	
Mean Length	827.604	

**Table 2:** Potential serine protease genes of *S. litura* midgut in response to the ingestion of Vip3Aa toxin.

WL-vs-M^1^			Unigene ID	nr_annotation	MC-vs-MVT^2^		
Regulated	log2FC	FDR			FDR	log2FC	Regulated
up	4.7040	4.46E-09	**c19837**	serine protease 36 [Mamestra configurata]	0	5.8649	up
up	3.8193	1.89E-08	**c19623**	serine protease 62 [Mamestra configurata]	0	3.9953	up
up	3.2438	1.15E-06	**c17572**	serine protease 54 [Mamestra configurata]	–	–	–
up	2.9806	2.71E-05	**c18669**	serine protease 37 [Mamestra configurata]	0	2.7062	up
up	2.7414	5.32E-05	**c14961**	serine protease 1 [Mamestra configurata]	–	–	–
up	2.6627	0.00042	**c23985**	serine protease 37 [Mamestra configurata]	–	–	–
up	2.4466	0.000986	**c11259**	serine protease 40 [Mamestra configurata]	0	−2.5298	down
up	2.4116	0.000661	**c21832**	serine protease 1 [Mamestra configurata]	0	−3.7417	down
up	2.4108	0.000949	**c17613**	serine protease 37 [Mamestra configurata]	2.73E-07	1.2901	up
up	2.2285	0.006531	**c19310**	serine protease 3 [Lonomia obliqua]	0	−2.5281	down
up	2.1944	0.004283	**c19576**	serine protease 1 [Mamestra configurata]	0.000445	1.0116	up
up	4.5718	2.00E-11	**c20161**	trypsinogen [Helicoverpa punctigera]	0	2.3939	up
up	3.9148	2.79E-09	**c19094**	trypsin-like serine protease [Spodoptera litura]	0	2.4131	up
up	3.5502	2.94E-06	**c6150**	trypsin-like serine protease [Spodoptera litura]	–	–	–
up	3.5361	1.07E-06	**c11399**	trypsin-like protease [Helicoverpa armigera]	–	–	–
up	3.0159	6.21E-06	**c19675**	trypsin precursor Hz19 [Helicoverpa zea]	–	–	–
up	2.9418	1.32E-05	**c19527**	PREDICTED: trypsin, alkaline C-like [Bombyx mori]	–	–	–
up	2.8859	3.59E-05	**c19330**	trypsin-like serine protease 9 [Ostrinia nubilalis]	2.48E-10	−1.4885	down
up	2.8332	0.00013	**c18929**	trypsin [Helicoverpa armigera]	0	−2.5266	down
up	2.7896	3.64E-05	**c21150**	PREDICTED: trypsin-1-like [Bombyx mori]	–	–	–
up	2.7312	0.000566	**c5682**	trypsin-like protease [Helicoverpa armigera]	0	2.5265	up
up	2.6323	0.001288	**c19277**	trypsin-like protease [Helicoverpa armigera]	0	2.7106	up
up	2.6305	0.000135	**c20871**	trypsin-like serine protease [Spodoptera litura]	–	–	–
up	2.6298	0.000217	**c11401**	PREDICTED: LOW QUALITY PROTEIN: trypsin CFT-1-like [Bombyx mori]	–	–	–
up	2.5242	0.00156	**c18046**	trypsin-like protease [Helicoverpa armigera]	1.50E-05	1.2165	up
up	2.1932	0.005754	**c19037**	trypsin [Heliothis virescens]	3.73E-07	1.2887	up
up	4.6265	1.74E-09	**c25196**	chymotrypsin-like protein 2 [Spodoptera litura]	–	–	–
up	3.7952	8.75E-06	**c25963**	chymotrypsin-like protein 2 [Spodoptera litura]	1.33E-09	−2.9884	down
up	3.7516	0.000108	**c20707**	chymotrypsin-like protein 2 [Spodoptera litura]	0	5.0745	up
up	3.1787	6.98E-05	**c20135**	chymotrypsin, partial [Heliothis virescens]	0	5.0409	up
up	2.5806	0.00031	**c19121**	chymotrypsin-like protein 2 [Spodoptera litura]	1.73E-05	−1.1248	down
up	2.5024	0.000657	**c19149**	chymotrypsin-like protease [Helicoverpa armigera]	–	–	–
up	2.1547	0.003726	**c19321**	chymotrypsin-like protein precursor [Spodoptera litura]	0	2.7072	up
up	2.0730	0.00687	**c20751**	chymotrypsin-like serine protease 14 [Ostrinia nubilalis]	–	–	–

^1^WL-vs-M: whole larvea vs. midgut.

^2^MC-vs-MVT: midgut control vs. midgut of larvea with vip3Aa treatment.
