# Supplementary material for: Transcriptional profiling analysis of Spodoptera litura larvae challenged with Vip3Aa toxin and possible involvement of trypsin in the toxin activation
Source: Sci Rep. 2016 Mar 30;6:23861. doi: 10.1038/srep23861 (PMC4812304; doi:10.1038/srep23861)
Supplement: Supplementary Information [file srep23861-s1.pdf]

Transcriptional profiling analysis of *Spodoptera litura* larvae challenged with Vip3Aa toxin and possible involvement of trypsin in the toxin activation

Feifei Song[1], Chen Chen[1], Songqing Wu[1], Ensi Shao[1], Mengnan Li[1], Xiong Guan[1], Zhipeng Huang[1,]*

[1]Key Laboratory of Biopesticide and Chemical Biology, Ministry of Education, Fujian Agriculture and Forestry University, 350002 Fuzhou, Fujian, People's Republic of China

*Corresponding author: zhipengh6841@163.com

Telephone number: 86-0591-83789258

Fax number: 86-0591-83789259

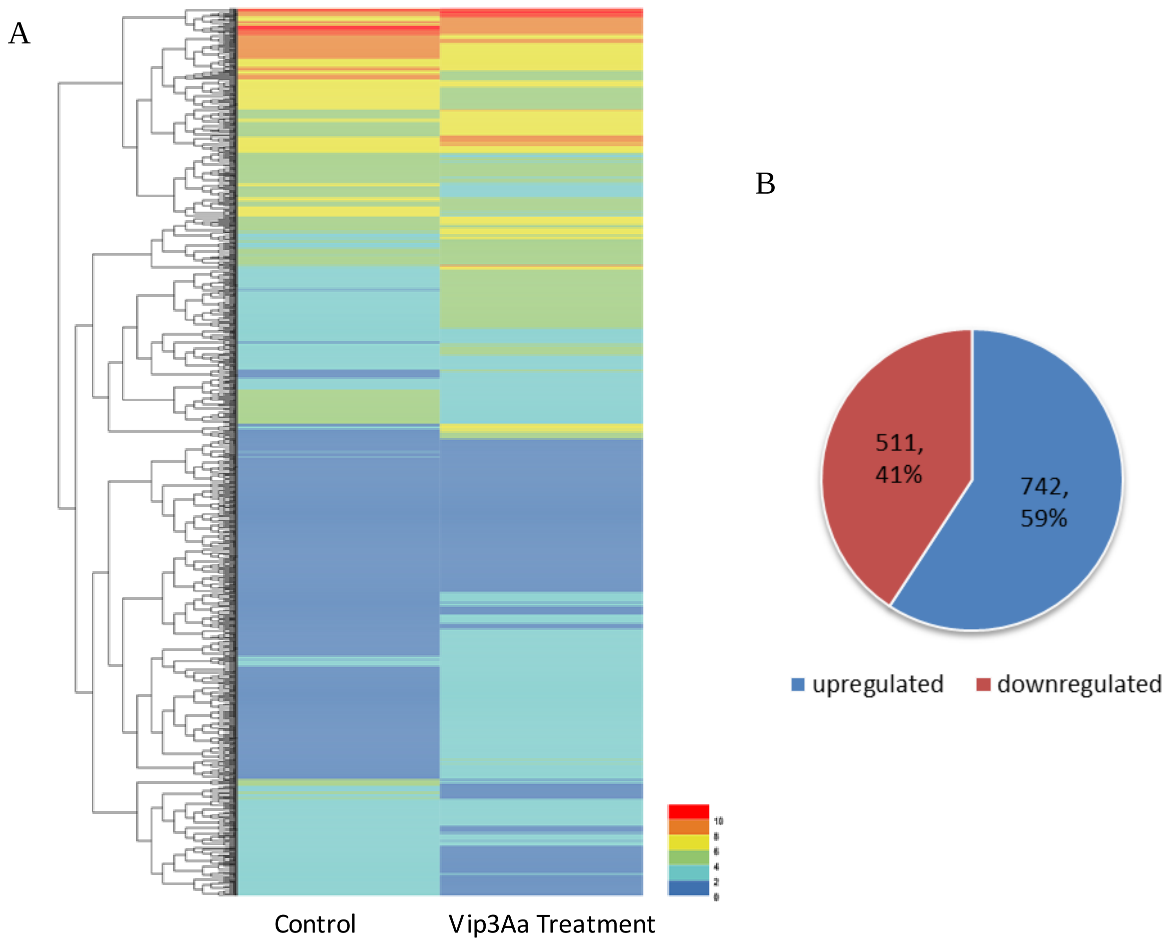

Figure S1. Differentially regulated genes of *S. litura* midgut during Vip3Aa intoxication. (A) Clustering analyses of DEGs after Vip3Aa intoxication. Each column represents a treatment, and each row represents a unigene. (B) Numbers of DGE unigenes of control and Vip3Aa-treated *S. litura*.

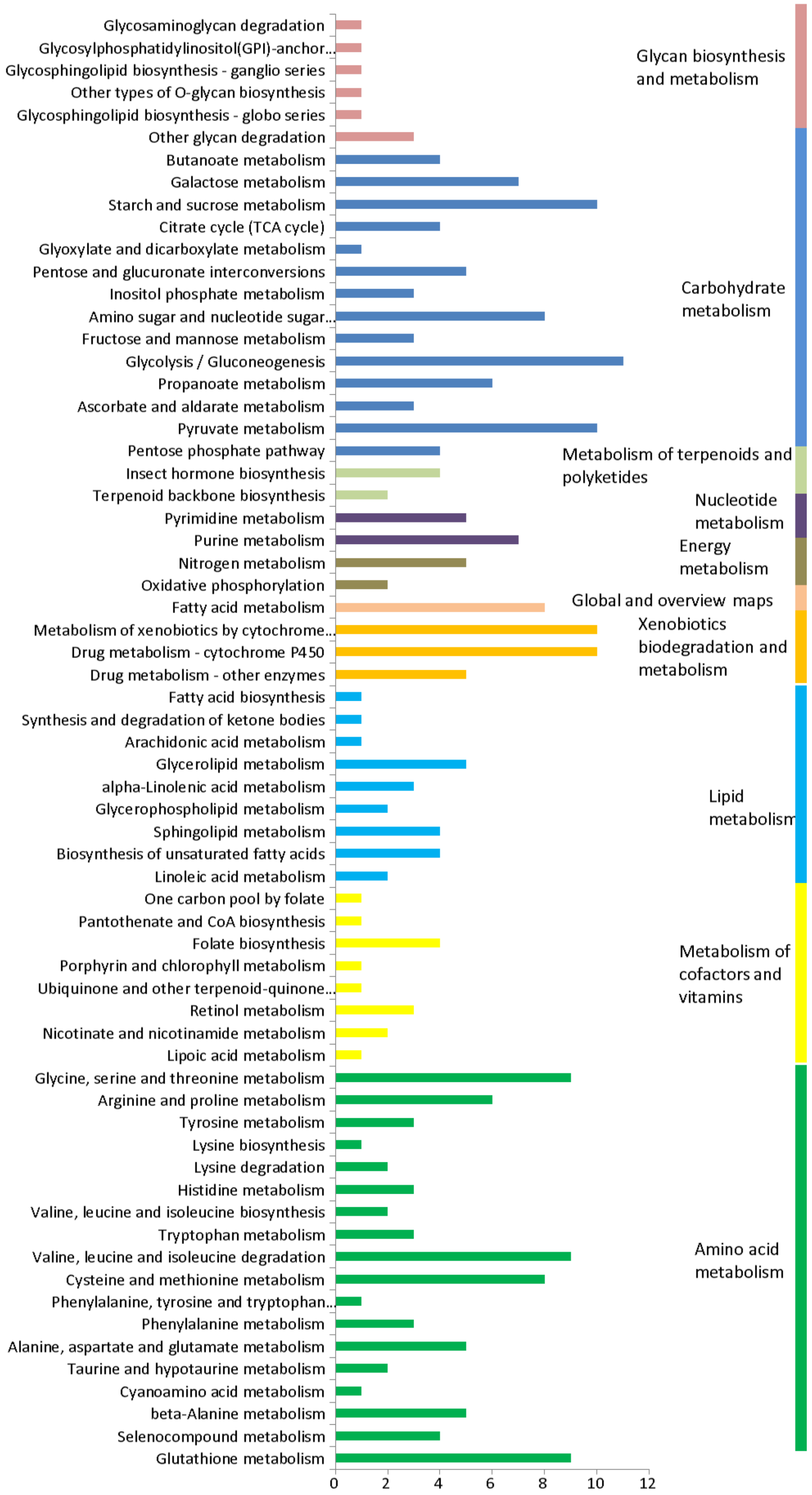

Figure S2 Summary of metabolism unigenes of *S. litura* larvae in response to the ingestion of Vip3Aa toxin.

Table S1 Immune-related unigenes of *S. litura* larvae in response to the ingestion of Vip3Aa toxin.

Function	Gene name	Unigene ID	nr_annotation	FDR	log2FC	regulated
Recognition	PGRP	c11677	PREDICTED: peptidoglycan recognition protein 3-like [Bombyx mori]	0.000509	-1.07437	down
		c21896	peptidoglycan recognition protein C [Helicoverpa armigera]	3.33E-16	-1.9233	down
		c25233	peptidoglycan recognition protein D [Ostrinia nubilalis]	1.28E-13	-2.65509	down
		c20498	peptidoglycan recognition protein B [Helicoverpa armigera]	0	-3.96387	down
	Scavenger receptor	c6828	PREDICTED: similar to scavenger receptor cysteine-rich protein isoform 1 [Tribolium castaneum]	1.93E-07	2.992627	up
		c20428	scavenger receptor class B member 3 [Bombyx mori]	3.55E-06	1.191757	up
		c19788	PREDICTED: similar to scavenger receptor cysteine-rich protein isoform 1 [Tribolium castaneum]	0.000173	1.0774	up
	lectin	c19357	C-type lectin [Heliothis virescens]	2.11E-14	1.768272	up
		c21695	C-type lectin 21 [Danaus plexippus]	2.60E-06	1.725227	up
		c15770	C-type lectin - mannose binding [Papilio xuthus]	6.75E-05	1.632966	up
	ßGRP	c5734	beta-1,3-glucan recognition protein 1 [Helicoverpa armigera]	0.000137	1.181256	up
	hemolin	c21312	hemolin [Helicoverpa zea]	0	4.85713	up
Transduction	Toll pathway	c18385	hypothetical protein KGM_03967 [Danaus plexippus]	8.90E-05	1.305731	up
	JAK-STAT pathway	c23114	hypothetical protein KGM_14365 [Danaus plexippus]	0.004367	1.18773	up
		c5690	PREDICTED: tyrosine-protein phosphatase corkscrew-like [Bombyx mori]	3.15E-05	1.121856	up
		c17585	Tyrosine-protein phosphatase non-receptor type 11 [Harpegnathos saltator]	6.95E-06	-1.20448	down
		c8425	pi3k [Bombyx mori]	5.46E-07	-1.29876	down
	MAPK	c18622	PREDICTED: torso-like protein-like [Bombyx mori]	6.10E-08	1.590182	up
		c17585	Tyrosine-protein phosphatase non-receptor type 11 [Harpegnathos saltator]	6.95E-06	-1.20448	down

Melanization	serpin	c8129	serine protease inhibitor [Papilio xuthus]	0	2.528995	up
		c23321	serine protease inhibitor 32 [Danaus plexippus]	2.45E-11	2.280243	up
		c20956	putative serpin [Hyphantria cunea]	0.000251	1.127645	up
	PPAE	c20351	prophenol oxidase activating enzyme 1 [Spodoptera litura]	3.64E-05	1.171194	up
Effector	diapausin	c17679	diapausin precursor [Spodoptera litura]	0	3.160039	up
	lysozyme	c20324	lysozyme-like protein 1 [Antheraea mylitta]	5.27E-05	1.166284	up
	defensin	c20369	defensin precursor [Spodoptera frugiperda]	0.004382	1.166916	up
Immune response	Hdd23	c21044	immune-related Hdd23 [Hyphantria cunea]	0.006028	1.473702	up
	Hdd1	c23168	Hdd1-like protein [Trichoplusia ni]	0	4.114703	up
		c22806	Hdd1-like protein [Danaus plexippus]	1.98E-13	3.022464	up

PGRP= Peptidoglycan recognition protein; ßGRP= β glucan recognition protein; PPAE= Prophenol oxydase activating enzyme.

Table S2 Unigenes homologous to the potentially Bt toxin receptors of *S. litura* larvae in response to the ingestion of Vip3Aa toxin.

Gene name	Unigene ID	nr_annotation	FDR	log2FC	regulated
ALP	c18356	alkaline phosphatase [Spodoptera exigua]	0	-1.9463	down
	c19507	PREDICTED: alkaline phosphatase, tissue-nonspecific isozyme-like [Bombyx mori]	0	-2.35337	down
	c19971	alkaline phosphatase 1 [Spodoptera litura]	0	-6.25675	down
	c19798	alkaline phosphatase 1 [Spodoptera litura]	0	-6.72673	down
G-protein[1]	c22536	PREDICTED: D(4) dopamine receptor-like [Bombyx mori]	0	1.985037	up
	c7152	PREDICTED: protein trapped in endoderm-1-like [Bombyx mori]	2.96E-14	2.008235	up
	c16418	neuropeptide receptor A17 [Bombyx mori]	4.08E-09	3.087901	up
	c16142	PREDICTED: alpha-1A adrenergic receptor-like [Bombyx mori]	0.001517	1.984716	up
cadherin	c17909	mutant cadherin [Helicoverpa armigera]	2.25E-07	1.570696	up
	c18147	PREDICTED: protocadherin-like wing polarity protein stan-like [Bombyx mori]	0.00113	-1.25067	down
Aminopeptidase N (APN)	c18685	midgut class 1 aminopeptidase N [Spodoptera exigua]	7.59E-08	-1.3676	down

1 annotating by GO database.

Table S3. Summary of 73 significantly differentially expressed transcripts from *S. litura* larvae midgut in response to the ingestion of Vip3Aa toxin.

**Unigene ID**	**nr_annotation**	**FDR**	**log2FC**	**regulated**
c12267	cytochrome CYP340K4 [Spodoptera littoralis]	2.85E-14	5.337373	up
c24505	cytochrome P450 [Spodoptera litura]	0	5.754862	up
c25622	cytochrome P450 [Helicoverpa armigera]	0	4.658589	up
c13139	putative cytochrome P450 [Danaus plexippus]	0	3.178457	up
c7824	cytochrome P450 CYP321A7 [Spodoptera frugiperda]	0	3.220674	up
c19112	PREDICTED: cytochrome b561 domain-containing protein 2-like [Bombyx mori]	0	2.127086	up
c17866	cytochrome P450 CYP4L4 [Mamestra brassicae]	8.48E-07	-1.250273	down
c23863	cytochrome P450 [Spodoptera litura]	1.24E-13	-2.53645	down
c20474	cytochrome P450 [Spodoptera litura]	0	-2.68906	down
c5076	high-affinity choline transporter [Trichoplusia ni]	4.43E-12	3.505538	up
c7083	inorganic phosphate transporter 1 [Bombyx mori]	0	2.546322	up
c14250	endonuclease-reverse transcriptase [Danaus plexippus]	0.000129	2.209259	up
c20410	PREDICTED: B(0,+)-type amino acid transporter 1-like [Bombyx mori]	0	2.142664	up
c20269	PREDICTED: B(0,+)-type amino acid transporter 1-like [Bombyx mori]	0	-2.31621	down
c10280	PREDICTED: facilitated trehalose transporter Tret1-1-like [Bombyx mori]	0	-2.63001	down
c4690	PREDICTED: facilitated trehalose transporter Tret1-like [Bombyx mori]	0	-2.77639	down
c25073	PREDICTED: monocarboxylate transporter 7-like [Bombyx mori]	9.09E-13	-2.43759	down
c14430	PREDICTED: monocarboxylate transporter 12-like [Bombyx mori]	0	-2.67101	down
c11964	PREDICTED: zinc transporter ZIP1-like [Bombyx mori]	0	-2.88921	down
c25542	sugar transporter [Danaus plexippus]	5.32E-13	-3.17118	down
c6659	sugar transporter [Danaus plexippus]	0	-3.68097	down
c18435	very high density lipoprotein [Helicoverpa zea]	0	-4.06635	down
c19573	very high density lipoprotein [Helicoverpa	0	-4.09206	down

	zea]			
c6015	very high density lipoprotein [Helicoverpa zea]	0	-4.37953	down
c22965	antennal esterase CXE14 [Spodoptera littoralis]	0	4.883817	up
c9702	antennal esterase CXE7 [Spodoptera littoralis]	3.33E-13	3.05433	up
c22497	chlorophyllide A binding protein precursor [Bombyx mori]	2.98E-09	-2.95125	down
c13497	chlorophyllide A binding protein precursor [Danaus plexippus]	8.80E-10	-3.73907	down
c20168	juvenile hormone binding protein an-0128 precursor [Bombyx mori]	0	2.565086	up
c20974	juvenile hormone diol kinase [Antheraea yamamai]	0	-5.4026	down
c16299	polycalin [Helicoverpa armigera]	0	-3.93479	down
c15680	polycalin [Trichoplusia ni]	9.99E-16	-2.97669	down
c5571	PREDICTED: ATP synthase subunit gamma, mitochondrial-like [Bombyx mori]	4.41E-13	3.89079	up
c4599	H+ transporting ATP synthase subunit d [Danaus plexippus]	2.14E-08	2.389133	up
c20814	PREDICTED: heat shock protein Hsp-16.1/Hsp-16.11-like [Bombyx mori]	0	2.841917	up
c16441	PREDICTED: zinc finger MIZ domain-containing protein 1-like [Bombyx mori]	3.55E-15	2.891747	up
c24932	PREDICTED: zinc finger MYND domain-containing protein 10-like [Bombyx mori]	0	-6.67029	down
c16222	Vanin-like protein 1 [Danaus plexippus]	0	-2.05474	down
c19029	vanin-like protein 1 precursor [Papilio xuthus]	0	-2.41942	down
c25580	PREDICTED: paired box protein Pax-6-like [Bombyx mori]	0	6.773916	up
c4816	PREDICTED: tetraspanin-1-like [Bombyx mori]	0	5.565138	up
c23506	PREDICTED: solute carrier family 23 member 2-like [Bombyx mori]	0	5.169746	up
c19861	PREDICTED: spidroin-1-like [Bombyx mori]	0	5.083898	up
c10167	putative fruitless [Danaus plexippus]	0	4.883817	up
c12275	putative kelch-like 10 [Danaus plexippus]	1.60E-08	4.665885	up
c25411	peritrophin-like protein [Spodoptera litura]	0	4.658589	up

c21060	PREDICTED: formin-like protein 3-like [Bombyx mori]	0	4.628245	up
c19188	chemosensory protein [Papilio xuthus]	0	4.453685	up
c21721	PREDICTED: prostaglandin reductase 1-like [Bombyx mori]	0	4.369457	up
c6753	PREDICTED: SOX domain-containing protein dichaete-like [Bombyx mori]	7.03E-14	3.965614	up
c29508	PREDICTED: UPF0704 protein C6orf165 homolog [Bombyx mori]	3.62E-09	3.906445	up
c6816	insulin-related peptide binding protein [Spodoptera frugiperda]	0	3.667266	up
c21029	ecdysteroid 22-kinase [Danaus plexippus]	0	3.519465	up
c16125	PREDICTED: nose resistant to fluoxetine protein 6-like [Bombyx mori]	0	3.358801	up
c12322	RecName: Full=Seroin; AltName: Full=Silk 23 kDa glycoprotein; Flags: Precursor	6.22E-06	3.225503	up
c24576	PREDICTED: homeobox protein orthopedia-like [Bombyx mori]	8.45E-12	3.045581	up
c6828	PREDICTED: similar to scavenger receptor cysteine-rich protein isoform 1 [Tribolium castaneum]	1.93E-07	2.992627	up
c9909	PREDICTED: mucin-3A-like [Bombyx mori]	1.24E-09	2.55012	up
c22229	proteophosphoglycan ppg4 [Leishmania braziliensis MHOM/BR/75/M2904]	0	2.436892	up
c6587	PREDICTED: T-related protein-like [Bombyx mori]	8.64E-05	2.431688	up
c19618	PREDICTED: tubulin beta chain-like [Aplysia californica]	1.12E-13	2.424683	up
c23038	wing disc-specific protein [Bombyx mori]	3.60E-08	2.399346	up
c19079	juvenile hormone epoxide hydrolase [Spodoptera exigua]	0.000488	-1.02732	down
c11508	TRAS3 [Bombyx mori]	0	-2.62016	down
c21887	gst1 [Spodoptera litura]	0	-2.93568	down
c19031	apolipophorin precursor protein [Bombyx mori]	0	-2.98517	down
c18795	REPAT1 [Spodoptera littoralis]	0	-3.04743	down
c15714	PREDICTED: synaptic vesicle glycoprotein 2A-like [Bombyx mori]	0	-3.58343	down
c12563	hypothetical protein KGM_15941 [Danaus plexippus]	0	-5.36763	down
c11312	PREDICTED: tubulin glycylase 3A-like [Bombyx mori]	0	-7.3459	down

c17167	serine protease 33 [Mamestra configurata]	8.69E-07	1.293962	up
c18827	trypsin precursor AiT6 [Agrotis ipsilon]	1.08E-05	-1.19949	down
c6408	PREDICTED: ATP-binding cassette sub-family G member 1-like [Bombyx mori]	5.52E-09	-1.39692	down

Table S4. Primers used for qRT-PCR amplifications.

#ID		Primer
c18046	Forward	TCGCATCATGGGTGAATGAA
	Reverse	GGTGTAGAAGAAGCAGGTGTAG
c18827	Forward	GTCATCATCCTCTTAGCCCTTT
	Reverse	ATGTTGGACATGTACGGGTATT
c18685	Forward	TTCCACTCCGCCTACTACTATC
	Reverse	GTAGGGTCAGAGAGAGGTTCTT
c19079	Forward	GGCAAGCAGGACAGTTCTAT
	Reverse	CGGTGGTTCTTTAGACGTAGTT
c17866	Forward	GATGAGAGCGTGAGAGAAGAAG
	Reverse	GACAGACAGTAGAGTGCGTATG
c18356	Forward	CGACGTAACTTTGTACCCGATAA
	Reverse	CCTCGGTCTTCCTTGTCTTTATT
c20814	Forward	GCCGTATTGGTTTCGTCATCT
	Reverse	GAAGAACGGGTCCCTCATAAAC
c20324	Forward	GTGAAGCACTGCTAGATGAAGA
	Reverse	GCCTCCCATTTCGACCAATA
c6408	Forward	AGCAACGCTGGCTGTATATT
	Reverse	TCTCACAAACACGCTCATCTC
c4599	Forward	CGCTGAGGTGGACAAAGAAT
	Reverse	GCACCGTTTCCATCGTCATA
c16418	Forward	AGACCCACACACGTCATTATC
	Reverse	GCTCGGTTGGAGTTTCTTCT
c12563	Forward	GGAGCTGATGCAGTGATTCT
	Reverse	GGAGTTCCATATCTTGGGTCTATG
β-actin	Forward	TGAGACCTTCAACTCCCCCG
	Reverse	GCGACCAGCCAAGTCCAGAC
